# Enhanced Chitin Deacetylase Production Ability of *Rhodococcus equi* CGMCC14861 by Co-culture Fermentation With *Staphylococcus* sp. MC7

**DOI:** 10.3389/fmicb.2020.592477

**Published:** 2020-12-10

**Authors:** Qinyuan Ma, Xiuzhen Gao, Linna Tu, Qi Han, Xing Zhang, Yabo Guo, Wenqin Yan, Yanbing Shen, Min Wang

**Affiliations:** ^1^Key Laboratory of Industrial Fermentation Microbiology, Ministry of Education, Tianjin Key Lab of Industrial Microbiology, College of Biotechnology, Tianjin University of Science and Technology, Tianjin, China; ^2^School of Life Sciences, Shandong University of Technology, Zibo, China; ^3^School of Science, College of Science, Engineering and Health, RMIT University, Melbourne, VIC, Australia

**Keywords:** chitin deacetylase, co-culture fermentation, chitin, quorum sensing, transcriptome

## Abstract

Chitin deacetylase (CDA) can hydrolyze the acetamido group of chitin polymers and its deacetylated derivatives to produce chitosan, an industrially important biopolymer. Compared with traditional chemical methods, biocatalysis by CDA is more environment-friendly and easy to control. However, most reported CDA-producing microbial strains show low CDA producing capabilities. Thus, the enhancement of CDA production has always been a challenge. In this study, we report co-culture fermentation to significantly promote the CDA production of *Rhodococcus equi* CGMCC14861 chitin deacetylase (ReCDA). Due to co-culture fermentation with *Staphylococcus* sp. MC7, ReCDA yield increased to 21.74 times that of pure culture of *R. equi*. Additionally, the enhancement was demonstrated to be cell-independent by adding cell-free extracts and the filtrate obtained by 10 kDa ultrafiltration of *Staphylococcus* sp. MC7. By preliminary characterization, we found extracellular, thermosensitive signal substances produced by *Staphylococcus* that were less than 10 kDa. We investigated the mechanism of promotion of ReCDA production by transcriptomic analysis. The data showed that 328 genes were upregulated and 1,258 genes were downregulated. The transcription level of the gene encoding ReCDA increased 2.3-fold. These findings provide new insights into the research of co-culture fermentation for the production of CDA and quorum sensing regulation.

## Graphical Abstract

GRAPHICAL ABSTRACT

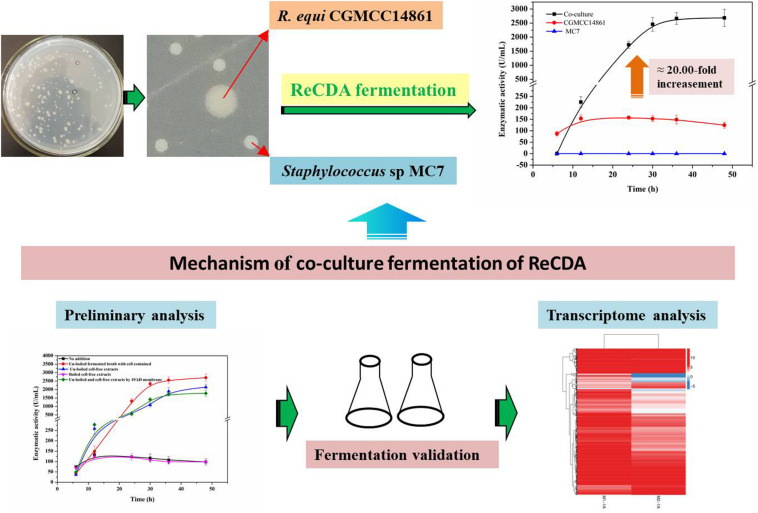

## Introduction

Chitin is the second most abundant biopolymer after cellulose, mainly obtained from waste products of the seafood industry, and is available at a relatively low cost (Grifoll-Romero et al., 2018). Chitosan is the deacetylated derivative of chitin (Raval et al., 2017), and it has gained broad interest in industrial applications owing to its unique features and physiological properties such as antimicrobial, antioxidant, and dilute acid-soluble property (Rinaudo, 2006; Younes and Rinaudo, 2015).

Currently, commercially available chitosan is still mainly produced by traditional chemical methods. However, they have many drawbacks such as being environmentally unsafe and poorly controlled in addition to the unstable nature of the products, especially in the deacetylation stage (Tsigos et al., 2000; Hamed et al., 2016). An enzymatic route employing microbial chitin deacetylase (CDA) can catalyze the deacetylation of chitin, and has been reported as an alternative for this process (Tsigos et al., 2000; Purushotham et al., 2012; Villa-Lerma et al., 2013; Raval et al., 2017). However, CDA is not commercially available due to the lack of potential microbial strains (Suresh et al., 2014).

As defined by Bader et al. (2010), co-culture means anaerobic or aerobic incubation of different specified microbial strains under aseptic conditions. Compared to pure cultures, co-culture fermentation can lead to better substrate utilization, increased productivity, increased adaptability to changing conditions, and resistance to contamination by unwanted microbes (Gutierrez-Correa et al., 1999; Alam et al., 2003). Co-culture fermentation has been widely used in many processes, including the production of vitamin C (Du et al., 2013), ethanol (Szambelan et al., 2004), acetic acid (Kondo and Kondo, 1996), amylase (Abate et al., 1999), inulinase (Öngen-Baysal and Sukan, 1996), xylanase (Juhász et al., 2003), nutraceuticals (Zhang et al., 2019), and so on. However, there are few reports on molecular mechanisms of co-culture effects in bacteria. Abate et al. (1999) reported a 2.5 fold increase in α-amylase production by mixed cultures of *Bacillus amyloliquefaciens* MIR-41 and *Zymomonas mobilis* Flo-B3 compared with pure cultures, which could be attributed to the lack of repression in the synthesis of α-amylase by ethanol and protease inhibition by the pH of the culture medium.

Previously, we isolated and identified a novel strain *Rhodococcus equi* CGMCC14861 that produces CDA (ReCDA) with the capability of hydrolyzing polymeric chitin substrates for the first time. In addition, the CDA gene from *Rhodococcus* genus was amplified for the first time (Ma et al., 2020a). Recently, we have shown the enzymatic properties of this ReCDA by phylogenetic analysis, biochemical characterization, and substrate spectrum determination (Ma et al., 2020b). The gene encoding ReCDA was 882 bp and located at 3,400 kb in the genomic map (Ma et al., 2020b). Phylogenetic analysis, biochemical characterization, and substrate spectrum determination have indicated the novelty of this newly identified ReCDA (Ma et al., 2020b). The rapid production of CDA by *R. equi* CGMCC14861 has advantages for industrial applications such as the recycling of the fermenters, bulky production, and cost-cutting (Ma et al., 2020a). Our recent reports have shown the enzyme proficiency of ReCDA using diverse chitin substrates (Ma et al., 2020b). However, further improvements in the fermentation performance based on *R. equi* CGMCC14861 should be conducted to obtain higher CDA yields. In the process of its isolation from soil samples, a smaller colony that could increase the ReCDA productivity of *R. equi* CGMCC14861 by co-culture fermentation was found accidentally and identified as *Staphylococcus* sp. MC7.

In the present work, a *Staphylococcus* strain was isolated and tested for production of ReCDA with *R. equi* CGMCC14861 during co-cultivation. It is worth noting that, to the best of our knowledge, this is the first report using a co-cultivation for ReCDA production. Preliminary characterization of inducers for quorum sensing (QS) and transcriptomic analysis was performed to understand the mechanism further. This study provides new insights into the research of co-culture fermentation for the production of CDA.

## Materials and Methods

### Materials and Strain

Chitin deacetylase producing strain *R. equi* CGMCC14861 was isolated in our lab and was stored at the China General Microbiological Culture Collection Center (Beijing, China). The model strain *Staphylococcus epidermidis* ATCC12228 was procured from Xinyang Zhongjian Metrology Biological Technology Co., Ltd. (Henan, China).

### Phylogenetic Analysis

The DNA of the identified candidate strain was extracted using TIANamp Bacteria DNA Kit (TIANGEN BIOTECH, Beijing, China) according to the manufacturer’s instructions. The 16S rDNA of the identified candidate strain was amplified using Taq DNA Polymerase (TaKaRa, Beijing, China) for colony polymerase chain reaction (PCR) under the following conditions: Each 20 mL PCR mixture contained 1× PCR buffer, 2.5 mM of dNTPs, 0.2 mM of 27F and 1,492R primers, 500 ng Template, and 0.5 units of Taq polymerase. The reaction conditions were 94°C for 5 min; 30 cycles at 94°C for 30 s, 52°C for 30 s, and 72°C for 90 s; and finally at 72°C for 10 min. The PCR products were purified using E.Z.N.A. Gel Extraction Kit (Omega Bio-tek, Guang zhou, China) according to the manufacturer’s instructions. Then, the purified PCR products were sequenced by Suzhou GENEWIZ Biotechnology Co., Ltd., (China), and the sequencing data were subjected to phylogenetic analysis. Phylogenetic trees were constructed using MEGA version 7.0 by neighbor-joining method with 1,000 bootstrap replicates (Kumar et al., 2016).

### Co-culture Conditions of *R. equi* CGMCC14861 and *Staphylococcus* sp. MC7

The starter cultures of *Staphylococcus* sp. MC7 and *R. equi* CGMCC14861 cultured in LB broth at 37°C and 200 rpm for 24 h were mixed in different ratios (the ratio of *Staphylococcus* sp. MC7 or *S. epidermidis* ATCC12228 to *R. equi* CGMCC14861 was 4:1, 3:2, 1:1, 2:3, 1:4, 1:9). The starter cultures of *S. epidermidis* ATCC12228 and *R. equi* CGMCC14861 cultured in LB broth at 37°C and 200 rpm for 24 h were mixed in 1:1. Then, the mixed cultures were incubated under the same conditions for 48 h. The total inoculation amount reached 10%, and the volume of medium was 50 mL in a 250 mL flask. The fermentation broth was taken from the main culture for the determination of its enzymatic activity at 6, 12, 24, 30, 36, and 48 h. Each experiment was performed in triplicate.

### Enzyme Assays

Bacterial cells obtained by centrifugal separation (12,000 rpm for 5 min) of 2 mL fermentation broth were washed and diluted with 0.2 M phosphate buffer (pH 7.0) to 2 mL, and then homogenized by grinding in a mortar and pestle in presence of liquid nitrogen. The enzymatic activity assay was performed as described previously (Sun et al., 2014). 4-Nitroacetanilide solution (0.3 mL, 200 mg/L) and 0.9 mL phosphate buffer (pH 7.0) was added to 0.3 mL crude ReCDA. The reaction mixture was incubated at 37°C for 1 h. One unit of CDA activity was defined as the amount of enzyme needed to catalyze the release of 1 μg 4-nitroaniline from 4-nitroacetanilide per hour.

### Scanning Electron Microscopy

Scanning electron microscopy (SEM) was performed as reported previously (Ong et al., 2016; Ma et al., 2020a) with the following modifications. *Staphylococcus* sp. MC7 was grown in LB liquid medium at 37°C for 24 h. The cultures were centrifuged at 8,000 rpm for 5 min, and the bacterial pellets were washed five times with phosphate-buffered saline (PBS). Samples were fixed overnight using 2.5% (v/v) glutaraldehyde, washed thrice with PBS, and then covered with a gold layer prior to observation using an SU1510 FE-SEM (Hitachi, Japan).

### ReCDA Production by *R. equi* CGMCC14861 Fermentation With Cell-Free Extracts of *Staphylococcus* sp. MC7

The starter cultures of *Staphylococcus* sp. MC7 were cultured in 50 mL LB broth at 37°C and 200 rpm for 24 h. Then, the fermented broth was collected and pretreated under three different conditions, including: (1) centrifugation to obtain cell-free culture extract; (2) boiling for 5 min, followed by centrifugation; and (3) ultrafiltration using a 10 kDa membrane, followed by filtrate collection. Then, the collected 5 mL cell-free extract of *Staphylococcus* sp. MC7 and 5 mL starter cultures of *R. equi* CGMCC14861 were inoculated at the same time for cultivation under the same conditions for 48 h. To study the effects of addition of fermented broth of *Staphylococcus* sp. MC7 on ReCDA production, different volumes (1, 3, 5, 7 mL) of cell-free culture liquid of *Staphylococcus* sp. MC7 were added for ReCDA production. The crude enzyme activity of the strains was measured.

### Transcriptomic Analysis

Fermentation broth (5 mL) of *R. equi* CGMCC14861 with cell-free extract of *Staphylococcus* sp. MC7 was centrifuged, and the bacteria were collected. Then, the bacterial samples were sent to Shenzhen BGI Co., Ltd. (Guangdong, China) for further quality and quantity evaluation, total RNA extraction, and mRNA library construction and analysis. It included gene ontology (GO) and Kyoto encyclopedia of genes and genomes (KEGG) pathway analyses of differentially expressed genes (DEGs). Total RNA was extracted using the RiboPure Bacteria Kit (Ambion, Foster City, CA, United States) according to the manufacturer’s instructions. The total RNA sample concentration, RIN (RNA integrity number), 23S/16S ribosomal RNA, and size were assessed using Agilent 2100 Bioanalyzer (Agilent RNA 6000 Nano Kit) and the purity of the samples was tested by NanoDrop (NanoDrop, Madison, United States). DNA in RNA samples was removed using DNase I and rRNA was depleted with Ribo-Zero Magnetic Kit (Invitrogen, Carlsbad, CA, United States). The mRNA samples were then fragmented using fragmentation reagent. First-strand cDNA was generated using random primers by reverse transcription, followed by second-strand cDNA synthesis. The synthesized cDNA was subjected to end-repair and then 3′ adenylated. The PCR products were purified with XP beads, and dissolved in elution buffer (RB solution). Agilent 2100 Bioanalyzer was used to check the distribution of the fragment size of the libraries and real-time quantitative PCR (qPCR; TaqMan Probe) was used to quantify the library. The qualified libraries were constructed using cBot to generate the cluster on the flow cell and the amplified flow cell was used to sequence pair ends on Illumina HiSeq 2000 (Illumina, San Diego, CA, United States) following the manufacturer’s instructions. The transcriptome data was deposited in the NCBI SRA (National Center for Biotechnology Information Sequence Read Archive) database under the accession number PRJNA605575.

### Statistical Analysis

Each sampling dataset was repeated at least thrice. Statistical analysis was performed using a one-way ANOVA with Tukey’s test. Origin8.5 was used to draw the figure, and the experimental data were expressed as mean ± SD, with *p* < 0.05 as the standard for significant difference.

## Results

### Identification of *Staphylococcus* sp. MC7

**FIGURE 1:**
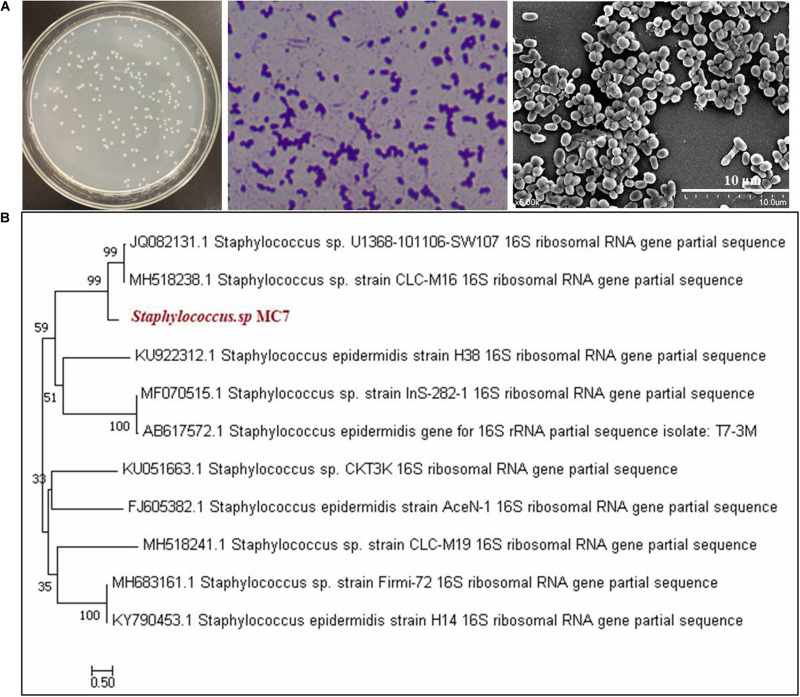
Morphological characteristics and phylogenetic analysis of *Staphylococcus* MC7 grown on Luria–Bertani (LB) agar plates at 37°C. **(A)** Colony morphology of strain MC7, gram staining, and scanning electron microscopy (SEM) image showing coccoid shape of this strain; **(B)** Phylogenetic tree of strain MC7 and related microorganisms collected at NCBI. The phylogenetic tree was constructed with the neighbor-joining method using MEGA 7.0 software. The numbers on the tree indicate the percentages of interior branch test sampling, derived from 1,000 replications.

As mentioned above, *Staphylococcus* sp. MC7 was found accidentally during the screening process of *R. equi* CGMCC14861. Its colony size was smaller than *R. equi* CGMCC14861, but the colony morphology was identical except for the colony color (Ma et al., 2020b). After incubation for 1–two days at 37°C, the colonies of strain MC7 appeared to be milky-white, round, smooth, opaque, glistening, gram-positive, non-motile, aerobic, and did not produce spores. Scanning electron microscopy images indicated MC7 to be coccoid with a diameter about 0.5 μm (Figure 1A). Alignment of 16S rDNA of the strain MC7 in GenBank database showed that it had 99% identity with several *Staphylococcus* strains (Figure 1B). Therefore, it was identified as *Staphylococcus* sp. MC7.

### Co-culture Fermentation at Different Incubation Ratios for ReCDA Production

**FIGURE 2:**
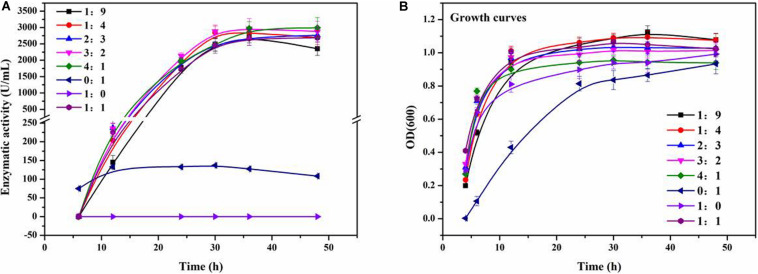
Co-culture fermentation of different mixing ratios of *R. equi* CGMCC14861 and *Staphylococcus* sp. MC7 for ReCDA (*R. equi* chitin deacetylase) production. **(A)** Enzymatic activity curve during co-culture fermentation; **(B)** Growth curves during co-culture fermentation.

In order to explore the effects of *Staphylococcus* sp. MC7 on the production of ReCDA by *R. equi* CGMCC14861, co-culture fermentation was performed with different inoculation ratios of *R. equi* CGMCC14861 and *Staphylococcus* sp. MC7. As shown in Figure 2A, when a pure culture of *Staphylococcus* sp. MC7 was used, no ReCDA activity was detected. When a pure culture of *R. equi* CGMCC14861 was used, the maximum activity of ReCDA was 157.61 U/mL. When the two strains were co-incubated in the ratio 4:1–1:9 (MC7:CGMCC14861), the enzymatic activity of ReCDA increased gradually and peaked at 30 h of fermentation (Figure 2A). When the mix incubation ratio was 4:1, the yield of ReCDA reached 2,974.05 ± 208.33 U/mL at maximum [the type of confidence interval used was standard deviation (SD)], but the biomass was the lowest under different co-cultivation conditions (Figure 2B).

### Preliminary Analysis of the Active Substance Produced by *Staphylococcus* sp. MC7

**FIGURE 3:**
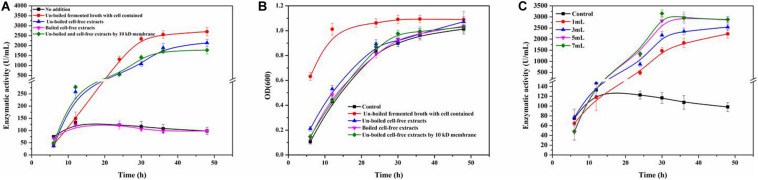
*Rhodococcus equi* CGMCC14861 chitin deacetylase activity and growth curve after addition of culture broth-containing *Staphylococcus* sp. MC7. **(A)** Crude ReCDA production under different fermentation conditions; **(B)** Growth curve under different fermentation conditions. **(C)** Crude ReCDA production during fermentation with different volumes of cell-free extracts of *Staphylococcus* sp. MC7.

Figure 3A shows the enzymatic activity of ReCDA during fermentation of *R. equi* CGMCC14861 with cells of *Staphylococcus* sp. MC7 and different types of cell-free extracts. It was found that when cell-free extracts without boiling were added, ReCDA production showed similar rising tendency during fermentation, and the yield of ReCDA reached a maximum of 2,141.35 ± 175.30 U/mL at 48 h. When the fermented broth of MC7 with cells were added, the yield of ReCDA was 2,703.14 ± 215.13 U/mL at 48 h. Different volumes of cell-free extracts without boiling of *Staphylococcus* sp. MC7 were added for ReCDA production and the addition had a significant effect on promotion of ReCDA production (Figure 3C). With the increase in amount added, the maximum production of ReCDA increased from 2,237.17 ± 195.11 U/mL to 3,153.90 ± 161.49 U/mL. There was no significant change in the biomass under different co-cultivation conditions except for the addition of untreated fermented broth-containing cells (Figure 3B).

**FIGURE 4:**
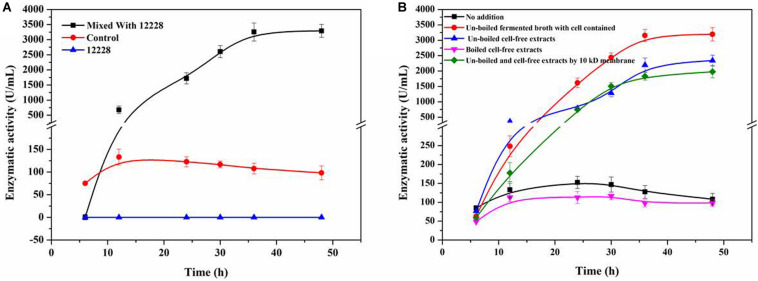
Co-culture fermentation of *R. equi* CGMCC14861 and *S. epidermidis* ATCC12228 for ReCDA production. **(A)** ReCDA production curves for co-culture fermentation with mixing ratio 1:1 of *R. equi* CGMCC14861 and *S. epidermidis* ATCC12228. **(B)** Crude ReCDA production under different fermentation conditions with *S. epidermidis* ATCC12228.

Figure 4A shows the co-culture fermentation process of *R. equi* CGMCC14861 with another *Staphylococcus* strain, *S. epidermidis* ATCC12228. Figure 4B shows the CDA activity during fermentation of *R. equi* CGMCC14861 with different types of cell-free extracts and with cells of *S. epidermidis* ATCC12228. During fermentation of *R. equi* CGMCC14861 with cell and cell-free extracts of *S. epidermidis* ATCC12228, the enzymatic activity of CDA showed similar results with that of *Staphylococcus* sp. MC7. As can be seen in Figure 4A, the crude enzyme activity of ReCDA in the broth was almost 3,500 U/mL at maximum, which was 24.75-fold higher than that in pure culture. When cell-free extracts of the two *Staphylococcus* strains were boiled, as shown in Figures 3A, 4B, there was no improvement in ReCDA production. When untreated cell-free extracts of *Staphylococcus* sp. MC7 and *S. epidermidis* ATCC12228 (without filtration) were added for the production of ReCDA, the crude enzyme activity increased to 2,341.35 ± 175.13 U/mL and 3,193.14 ± 215.39 U/mL, respectively, at 48 h (Figures 3A, 4B). The filtrate collected by 10 kDa ultrafiltration tube showed the same effects of enhancement on the production of ReCDA (Figures 3A, 4B).

### Transcriptomic Analysis of Co-culture Fermentation for ReCDA Production

**FIGURE 5:**
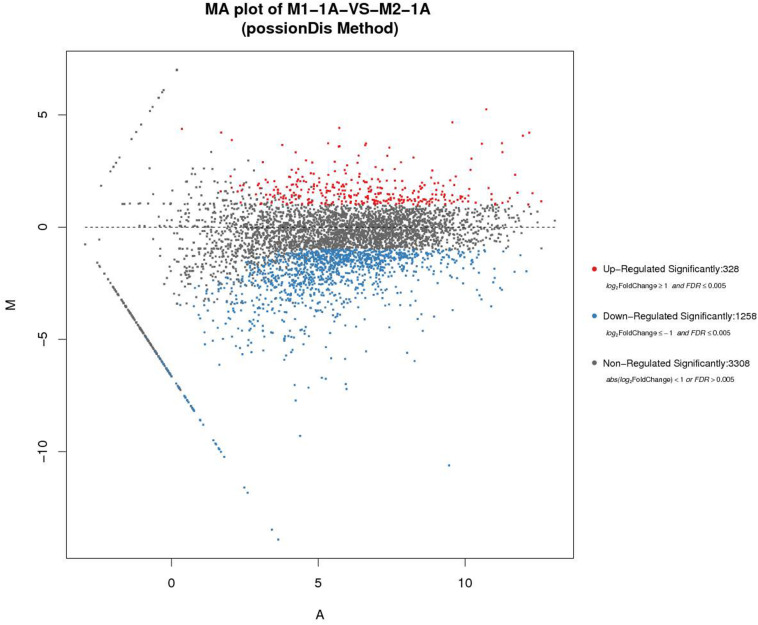
The comparison and analysis of transcriptomic data of the two samples. MA (Bland–Altman) plot of M1-1A: pure culture of *R. equi* CGMCC14861; M2-1A:co-culture of *R. equi* CGMCC14861. The horizontal axis represents the average expression level after logarithmic conversion, which represents the value of A. The larger the value, the higher the expression level. The vertical axis represents the difference multiple after logarithmic conversion, representing the M value. The greater absolute M value of a gene indicates greater difference in the expression level of the gene in the difference pair.

**FIGURE 6:**
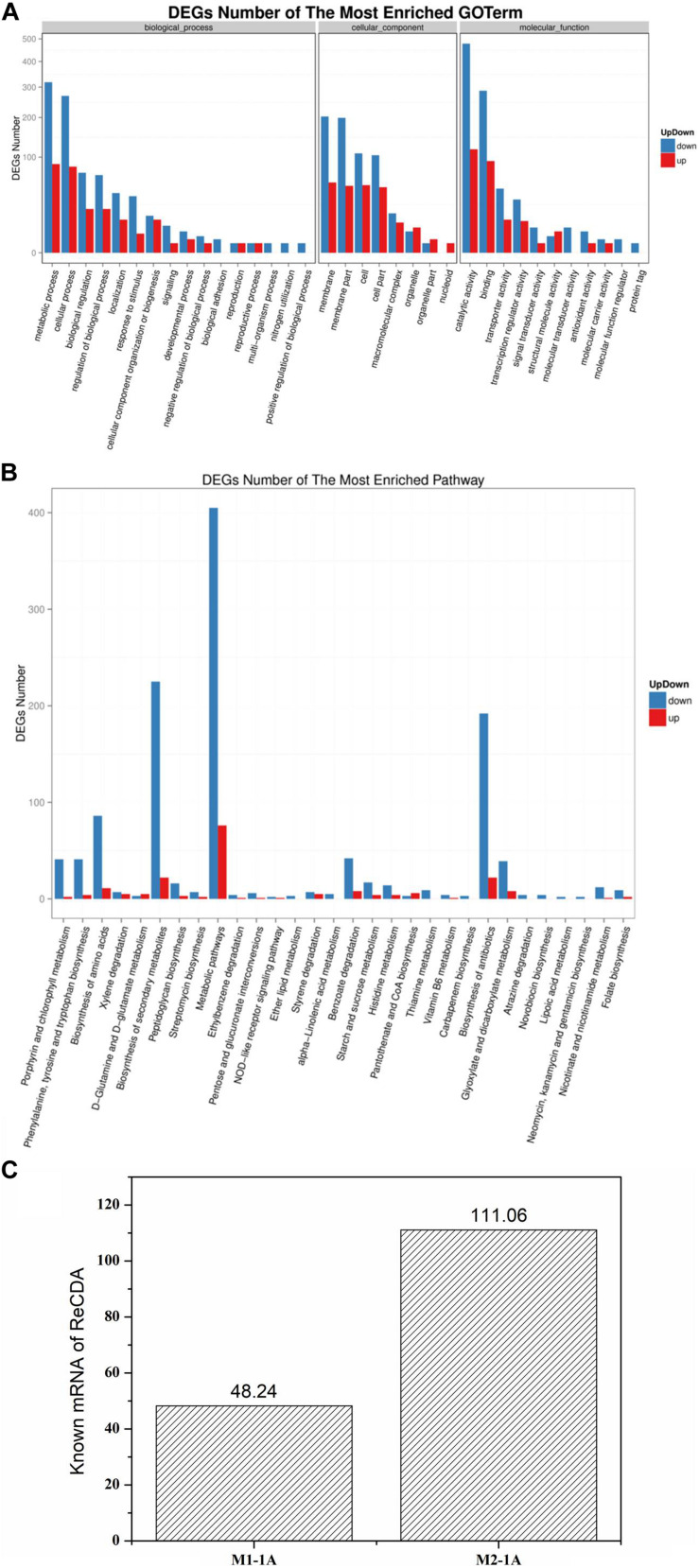
Gene ontology (GO) function and pathway analysis of significantly up- and downregulated differentially expressed genes (DEG) and the transcription level of ReCDA. **(A)** GO functional classification of significantly up- and downregulated DEGs. The horizontal axis represents GO terms, and the vertical axis represents the number of significantly up- and downregulated DEGs in the corresponding GO terms. **(B)** Kyoto encyclopedia of genes and genomes (KEGG) classification of significantly up- and downregulated DEGs. The horizontal axis represents the pathway enriched with DEGs (in order of significance of enrichment; significance of enrichment decreases successively from left to right), and the vertical axis represents the number of significantly up- and downregulated DEGs in the corresponding pathway. **(C)** Comparison of the transcription level of ReCDA-coded genes.

*Rhodococcus equi* CGMCC14861 cells collected from pure cultures (M1-1A) and cultures with the filtrate obtained by 10 kDa ultrafiltration of cell-free extracts of *Staphylococcus* sp. MC7 (M2-1A) were used for transcriptomic analysis. In total, 13,240,124 and 8,013,112 reads were uniquely mapped to the reference genome, accounting for 90.39% and 85.16% mapping rate for M1-1A and M2-1A, respectively. There were 1,586 DEGs, including 328 upregulated (accounting for 20.68%) and 1,258 downregulated (accounting for 79.32%) in M2-1A compared to the control M1-1A (Figure 5). The cluster heat map of significantly DEGs (intersection) is shown in Supplementary Figure 1. As per the results of GO function analysis of DEGs, 1,078 genes related to biological process, 857 genes related to cellular components, and 1,108 genes related to molecular function were expressed (Supplementary Figure 2A). As indicated in Figure 6A, the DEGs were mainly distributed in genes related to catalytic activity, binding, metabolic process, cellular process, and membrane, which accounted for 73.38% of the total number of DEGs. As per KEGG pathway analysis of DEGs (Supplementary Figure 2B), there were 219 DEGs involved in cellular community, membrane transport, and signal transduction. Except environmental information processing and cellular process-related genes, there were 88.10% DEGs involved in biochemical metabolic pathways. The main pathways involved were metabolic pathways, biosynthesis of secondary metabolites, and biosynthesis of antibiotics (Figure 6B). From Figure 6C, it can be observed that the transcription level of *ReCDA* gene in co-culture was 2.3-fold that in pure culture. qRT-PCR was performed to verify the results of RNA sequencing (RNA-Seq). As shown in Supplementary Figure 4, RNA transcription level of ReCDA in M2-1A was 1.9-fold greater than that in M1-1A.

## Discussion

The utilization of chitin is important for C and N recycling, which is an abundant biopolymer in nature. CDA can be used for deacetylation of chitin and its derivates. We have been in the process of exploring highly efficient strains for CDA production. *R. equi* CGMCC14861 was found to be a promising CDA producer in our previous study (Ma et al., 2020b). During the screening process from soil samples, a smaller colony that could increase the ReCDA production by *R. equi* CGMCC14861 by co-culture fermentation (Figure 2A) was identified. In this study, it was identified as *Staphylococcus* sp. MC7. In addition, the auxo-action role and preliminary mechanism of strain MC7 on the CDA production of *R. equi* CGMCC14861 were investigated.

### Co-culture Fermentation Enhanced ReCDA Production

As shown in Figure 2A, there was no significant difference in the yield of ReCDA in co-culture fermentation of *Staphylococcus* sp. MC7 and *R. equi* CGMCC14861 in different ratios. The yield of ReCDA in co-culture fermentation with 4:1 inoculation was at maximum 18.87-fold that in pure culture of *R. equi* CGMCC14861. The CDA productivity of *R. equi* CGMCC14861 in co-culture and other microorganisms in pure culture are reviewed in Table 1. It can be identified that the production of CDA in this study was the highest ever reported. Although co-culture fermentation has been utilized to produce enzymes, for example, laccase produced by *Trametes* sp. AH28-2 in co-cultivation with *Trichoderma* sp. ZH1 (Zhang et al., 2006), there have been no reports on CDA production by co-culture. Thus, this is the first report for the production of CDA by co-culture, and *Staphylococcus* sp. MC7 was found to be the first microorganism that could enhance the production of CDA by co-culture fermentation.

**TABLE 1:** Comparison of productivities of CDA (chitin deacetylase) by co-culture and pure culture fermentation.

**Methods**	**Time (h)**	**Volume of medium (mL/250 mL)**	**pH**	**Temperature (°C)**	**Enzymatic activity (U/mL)**	**References**
Relationship to cell growth rate in *Cunninghamella bertholletiae* IFM 46114	120	75	4.5	28	99.36	Amorim et al. (2005)
Statistical mutagenesis for *Penicillium oxalicum* ITCC 6965	90	50	8.0	30	1,747.08	Pareek et al. (2011)
Statistical optimization for *Rhodococcus erythropolis* HG05	60	40	7.0	30	238.89	Sun et al. (2014)
Addition of abscisic acid for *Colletotrichum gloeosporioides*	48	50	3.5	28	496.63	Ramos-Puebla et al. (2016)
This study	48	50	7.0	37	2,974.05	This work

### The Inducer Produced by *Staphylococcus* May Be Extracellular Peptide Less Than 10 kDa

As reported, the influence of co-cultivation may be realized by cell-to-cell interactions (Meyer and Stahl, 2003) or signal substances (Bader et al., 2010). In order to investigate the enhancement by *Staphylococcus* sp. MC7 on *R. equi* CGMCC14861 was dependent on cells or not, cell-free broth of *Staphylococcus* sp. MC7 cultured for 24 h treated by boiling or ultrafiltration was added into the fermentation medium of *R. equi* CGMCC14861 during inoculation. As shown in Figure 3A, compared to pure culture, addition of cell-free extracts of *Staphylococcus* sp. MC7 enhanced the production of ReCDA. Compared with addition of broth-containing cells, the maximum production of ReCDA was a little lower. This demonstrates that as long as living *Staphylococcus* cells exist in fermentation medium, the synergy will be improved. However, as also shown in Figure 3B, the enhancing effect was cell-independent. *Staphylococcus* sp. MC7 interacted with *R. equi* CGMCC14861 through extracellularly secreted chemical substances. The biomass of *R. equi* CGMCC14861 did not change with the addition of cell-free extracts of *Staphylococcus* sp. MC7 compared to the control (Figure 3B), which demonstrates that the enhancement of enzymatic activity did not depend on the growth phase of the strain, but on some simulating factors in the fermented broth.

In order to confirm whether the chemical substance from *Staphylococcus* sp. MC7 was specific to the strain or not, *Staphylococcus* sp. MC7 was replaced by *Escherichia coli*. BL21, *E. coli* DH5a, *Pichia pastoris* GS115 and *Bacillus subtili*s WB600. As shown in Supplementary Figure 3, the ReCDA activity was detected in the initial stage of the fermentation and was hardly detected later. Then *Staphylococcus* sp. MC7 was replaced by the model strain *S. epidermidis* ATCC12228, which showed over 99% similarity to *Staphylococcus* sp. MC7. As indicated in Figure 4A, *S. epidermidis* ATCC12228 showed the same effects of promoting ReCDA production as *Staphylococcus* sp. MC7. These results suggest that *S. epidermidis* ATCC12228 positively contributed to the increase in production of ReCDA. *S. epidermidis* ATCC12228 had a stronger ability of co-culture fermentation for ReCDA production than *Staphylococcus* sp. MC7. As indicated in Figure 3C, this enhancement was related to the amount of cell-free extract. Therefore, we speculated that *S. epidermidis* ATCC12228 may secrete more simulating factors than *Staphylococcus* sp. MC7.

As is known, QS is a chemical communication process, which may be inter- or intra-genus (Banerjee and Ray, 2017). The signal molecules of QS are usually acetylated homoserine lactone in gram-negative bacteria (Fuqua et al., 2001) or peptides in gram-positive bacteria (Shimkets, 1999). Therefore, we conclude that the inter-cell communication between *Staphylococcus* sp. MC7 and *R. equi* CGMCC14861 may have occurred. Interactions between species and genera are not rare. For example, co-cultivation with *Fusarium oxysporum* could trigger the *afp* gene of *Aspergillus giganteus* and improve the expression of the antifungal protein (Meyer and Stahl, 2003). In the *Staphylococcus* genus, QS is common. It was reported that the modified 46 amino acid peptide encoded by *argD* gene of *Staphylococcus* is the final autoinducing peptide (AIP; Ji et al., 1997). The mature AIP consists of seven–nine amino acids and contains a thiolactone or lactone ring between cystine and C-terminal (Ji et al., 1997). Therefore, in order to verify this, cell-free extracts of *Staphylococcus* sp. MC7 were treated by boiling and ultrafiltration and used as inducers of fermentation of *R. equi* CGMCC14861. From the results seen in Figures 3A, 4B, we conclude that the inducing metabolite produced by *Staphylococcus* was thermosensitive and less than 10 kDa, which is in agreement with a previous report (Bassler, 1999). During signal transduction in *Staphylococcus*, AIP interacts with the transduction system by binding to the histidine kinase sensor (Ji et al., 1997). Therefore, we hypothesized that some kinases that were widely present in *R. equi* CGMCC14861 may play a key role in the interaction between the two strains. Future investigations should be performed to explore the mechanisms in detail.

### Co-culture With *Staphylococcus* Upregulated the Gene Encoding ReCDA

Quorum sensing is a sequential process in bacteria (Banerjee and Ray, 2017). In order to understand the mechanism of co-culture of *Staphylococcus* sp. MC7 and *R. equi* CGMCC14861, transcriptomic analysis was performed. As shown in Figure 5, the co-culture resulted in different gene expression profiles. Particularly, most of DEGs were found to be downregulated, which may be due to the effects of toxins secreted by *Staphylococcus* sp. MC7 on the cell metabolism of *R. equi* CGMCC14861. This is consistent with the report on the DEGS in *Staphylococci*, which is related to the QS effect (Lin et al., 2013). According to previous study, in the QS circuit of gram-positive bacteria, an inducer molecule is transformed into cell membrane and interacts with a receptor protein (Banerjee and Ray, 2017). It is reported that QS-mediated gene expression also controls biofilm formation (Goo et al., 2015). Therefore, the DEGs involved in cellular community, membrane transport, and signal transduction may be related to the interactions between *Staphylococcus* and *R. equi* CGMCC14861. As shown in Figure 6A and Supplementary Figure 2B, most of the DEGs were related to catalytic activity, binding, metabolic process, cellular process, and membrane and 219 DEGs involved in cellular community, membrane transport, and signal transduction. Sun et al. (2015) reported that QS could regulate the chitin metabolism of *Vibrio cholerae* A1552, a chitinolytic bacterium. According to genome sequence and annotation, there was one CDA in *R. equi* CGMCC14861. Therefore, the transcription level of the gene encoding ReCDA was analyzed. The 2.3-fold improvement in transcription level of the ReCDA gene led to the enhanced production of ReCDA. However, in the future, it is important to explore the signal transduction process and the effects of biofilm formation on the production of ReCDA between the co-culture of *R. equi* CGMCC14861 and *Staphylococcus* sp.

In this work, an isolated strain *Staphylococcus* sp. MC7 was accidentally found to be beneficial for production of CDA by *R. equi* CGMCC14861. Compared to pure culture, co-culture fermentation with MC7 resulted in 21.74 times improvement in the yield of ReCDA, which is the highest level compared with the previous reports. The signal substances were analyzed preliminarily and found to be thermosensitive and less than 10 kDa, which may be a peptide secreted extracellularly by *Staphylococcus*. Transcriptomic analysis indicated that co-culture caused significantly different expression of genes in *R. equi* CGMCC14861. The transcription level of the gene encoding *ReCDA* was improved 2.3-fold. This work provides potential application and research value for co-culture fermentation, the production of CDA, and QS mechanism. According to the report results, kinases (Schlessinger, 2000; Boggon and Eck, 2004), transcription factor (Sharrocks, 2001), and regulatory proteins (Longo et al., 2016; Sumimoto et al., 2019) may have the positive effects on the improvement of ReCDA. Therefore, further studies on the kinases, transcription factor and regulatory proteins with molecular weight less than 10 kDa annotated in the genome of *S. epidermidis* ATCC12228 can be important for the ReCDA fermentation.

## Data Availability Statement

The datasets presented in this study can be found in online repositories. The names of the repository/repositories and accession number(s) can be found below: https://www.ncbi.nlm.nih.gov/, PRJNA605575.

## Author Contributions

QM and XG performed the research, analyzed the data, and wrote the manuscript. XZ, YG, and WY performed the research. LT and QH analyzed the data and provided technical assistance. MW designed the research. YS designed the research, analyzed the data, and wrote the manuscript. All authors read and approved the final manuscript.

## Conflict of Interest

The authors declare that the research was conducted in the absence of any commercial or financial relationships that could be construed as a potential conflict of interest.

## Abbreviations

ReCDA: *Rhodococcus equi* CGMCC14861 chitin deacetylase
DNB: DNA nanoball
SEM: scanning electron microscopy
DEG: differentially expressed genes
GO: gene ontology
KEGG: Kyoto encyclopedia of genes and genomes
QS: quorum sensing
BLAST: basic local alignment search tool
PBS: phosphate-buffered saline
LB: Luria–Bertani
RIN: RNA integrity number
RNA-Seq: RNA sequencing
AIP: autoinducing peptide.
