# Supplementary material for: Enhanced Chitin Deacetylase Production Ability of Rhodococcus equi CGMCC14861 by Co-culture Fermentation With Staphylococcus sp. MC7
Source: Front Microbiol. 2020 Dec 10;11:592477. doi: 10.3389/fmicb.2020.592477 (PMC7758288; doi:10.3389/fmicb.2020.592477)
Supplement: Supplementary file 1 [file Data_Sheet_1.PDF]

Prokaryotic Strand-Specific Transcriptome Library Construction Protocol (Illumina)

Document NO.:	SOP-SS-029
Version NO.:	A1
Effective Date:	2019-06-12

1 Introduction

Strand specific transcriptome research can determine whether a transcript comes from sense strand or antisense strand, can identify the boundary of transcript and more precisely the amount of transcripts. It provides an important approach for gene fine structure and gene expression regulation. Strand specific transcriptome library construction is completed by removing rRNA from total RNA, sequenced by Illumina high-throughput platform, and followed by bioinformatics analysis.

2 Experimental Procedures

2.1 The Method of Sample Detecting

By using Agilent 2100 Bioanalyzer the total RNA samples' concentration, RIN, 23S/16S and size would be detected. The purity of the samples was tested by NanoDrop™.

2.2 Library Construction

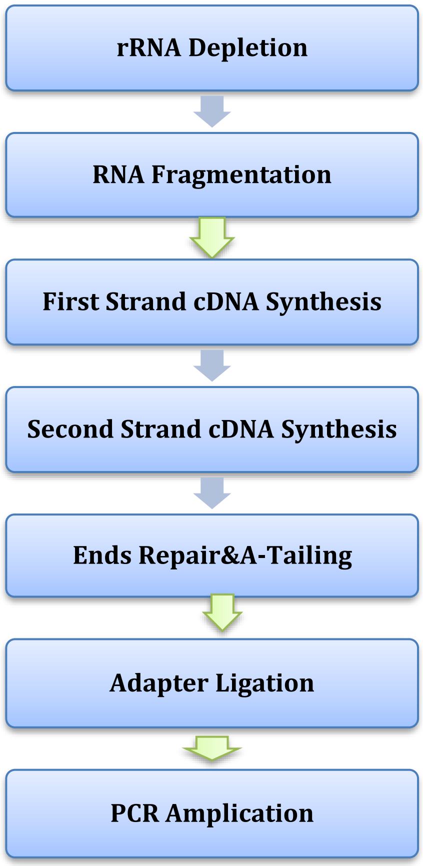

Fig1. Prokaryotic Strand-Specific Transcriptome Library Construction Workflow

2.3 DNase I Digest

DNase I degraded double-stranded and single-stranded DNA presenting in RNA samples.

华大基因 BGI

2.4 rRNA Depleted

Total RNA samples were treated with Ribo-Zero™ Magnetic Kit to deplete rRNA.

2.5 Fragmentation

RNA molecules were fragmented into small pieces using fragmentation reagent.

2.6 cDNA Synthesis

First-strand cDNA was generated using random primers reverse transcription, followed by a second-strand cDNA synthesis.

2.7 End Repair, Add A and Adaptor Ligation

The synthesized cDNA was subjected to end-repair and then was 3’ adenylated. Adapters were ligated to the ends of these 3’ adenylated cDNA fragments.

2.8 PCR

Several rounds of PCR amplification with PCR Primer Cocktail and PCR Master Mix are performed to enrich the cDNA fragments. Then the PCR products are purified with Ampure XP Beads.

2.9 Library Quality Control

The libraries were assessed quality and quantity in two methods: check the distribution of the fragments size using the Agilent 2100 bioanalyzer, and quantify the library using real-time quantitative PCR (QPCR) (TaqMan Probe).

2.10 Sequencing

The qualified libraries will amplify on cBot to generate the cluster on the flowcell. And the amplified flowcell will be sequenced pair end on the Illumina System.

---END---
