# Supplementary material for: Enhanced Chitin Deacetylase Production Ability of Rhodococcus equi CGMCC14861 by Co-culture Fermentation With Staphylococcus sp. MC7
Source: Front Microbiol. 2020 Dec 10;11:592477. doi: 10.3389/fmicb.2020.592477 (PMC7758288; doi:10.3389/fmicb.2020.592477)
Supplement: Supplementary file 2 [file Data_Sheet_2.DOCX]

Enhanced chitin deacetylase production ability of *Rhodococcus equi* CGMCC14861 by co-culture fermentation with *Staphylococcus* sp. MC7

Qinyuan Ma^1&2#^, Xiuzhen Gao^2#^, Linna Tu^1^, Qi Han^3^, Xing Zhang^1^, Yabo Guo ^1^, Wenqin Yan^1^, Yanbing Shen^1*^, Min Wang^1*^

^1^ Key Laboratory of Industrial Fermentation Microbiology (Tianjin University of Science &Technology), Ministry of Education, Tianjin Key Lab of Industrial Microbiology, College of Biotechnology, Tianjin University of Science and Technology, Tianjin 300457, P. R. China

^2^ School of Life Science, Shandong University of Technology, Zibo 255049, China

^3^ School of Science, College of Science, Engineering and Health, RMIT University, Melbourne, VIC, Australia

^#^These authors contributed equally to this work.

E-mail address: 18660686917@163.com (QY Ma); gaoxz@sdut.edu.cn (XZ Gao); cnhanqi@gmail.com (Q Han); tulinna79@163.com (LL Tu); 1262991609@qq.com (X Zhang); 1445805951@qq.com (YB Guo); 958417539@qq.com (WQ Yan); shenyb@tust.edu.cn (YB Shen); minw@tust.edu.cn (M. Wang).

*** Correspondence:**Yanbing Shen, Min Wang
E-mail: shenyb@tust.edu.cn; minw@tust.edu.cn.

Keywords: Chitin deacetylase, co-culture fermentation, chitin, quorum sensing, transcriptome

# Supplementary Fig. S1


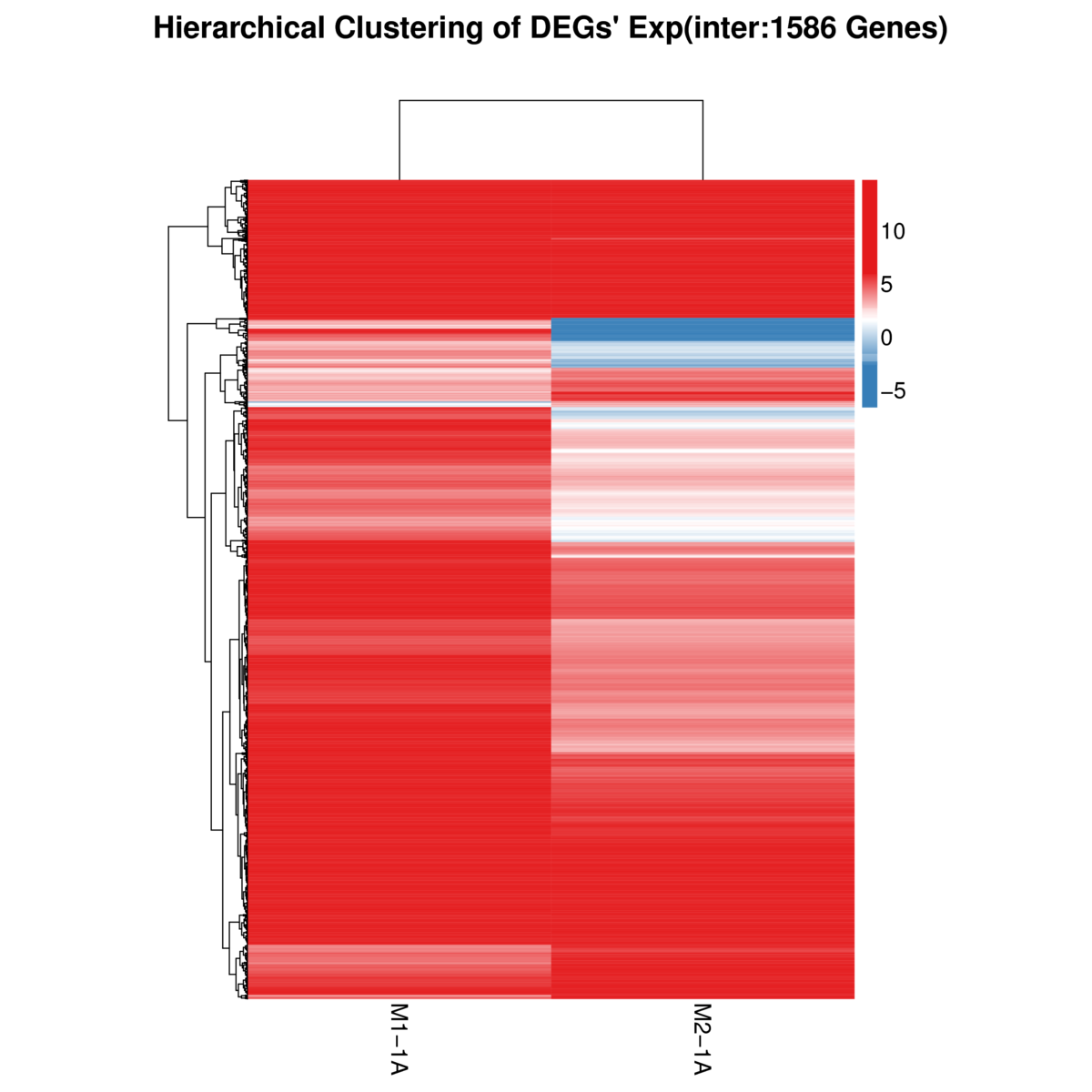


Hierarchical clustering of DEGs’ Expression (inter: 1586 genes). The vertical axis is the genes being expressed significantly differentially. Color represents expression after logarithmic conversion, and the greater the color difference, the greater the gene expression difference. Red is for genes highly expressed, and blue is for genes poorly expressed.

# Supplementary Fig. S2


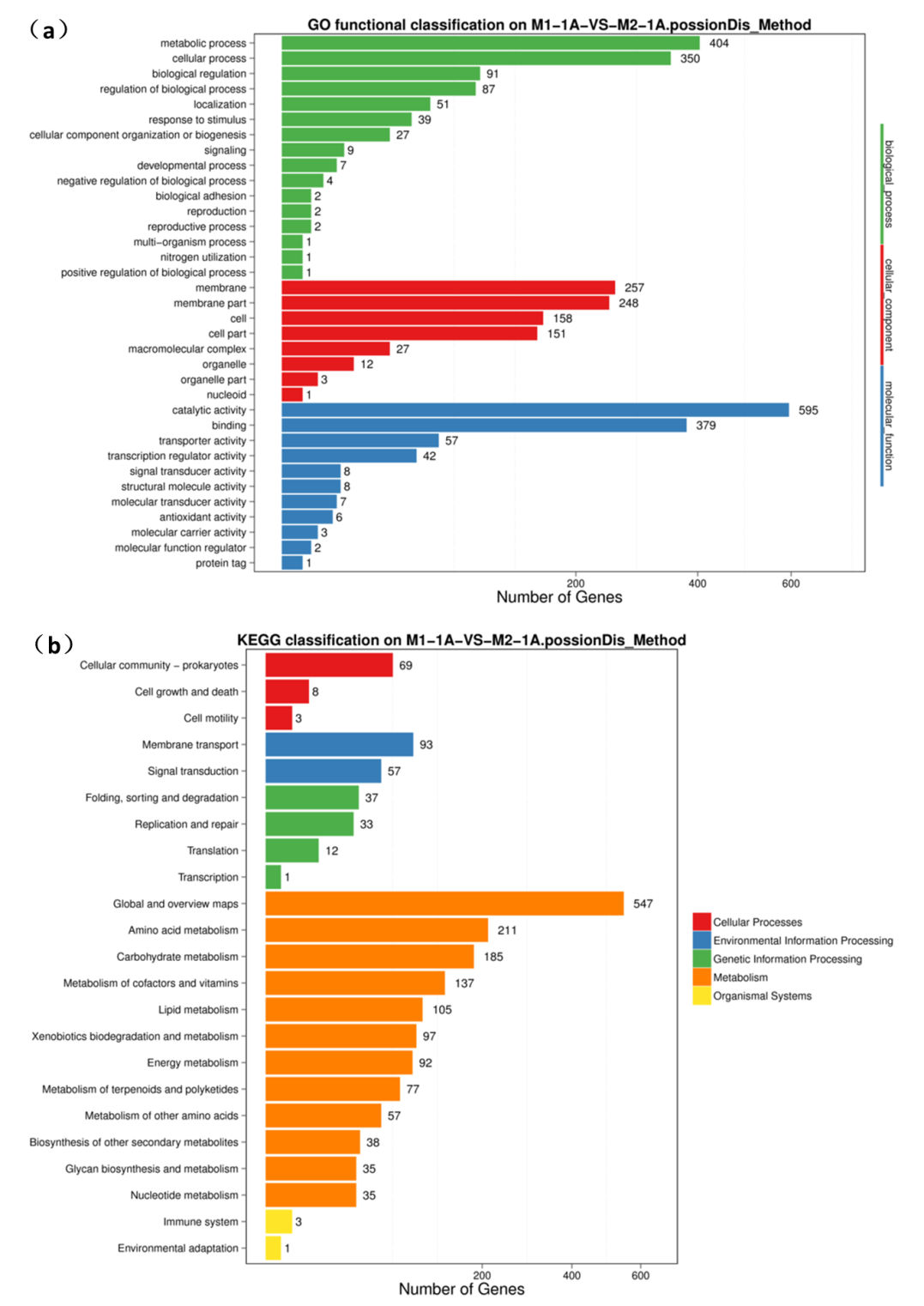


GO function and pathway analysis of differentially expressed genes. (a) GO functional classification on M1-1A-VS-M2-1A. The vertical axis is GO terms, and the horizontal axis is the number of genes being differentially expressed in the corresponding GO terms. (b) KEGG classification on M1-1A-VS-M2-1A. The horizontal axis is the number of genes being significantly differentially expressed, and the vertical axis is pathway classification.

# Supplementary Fig. S3


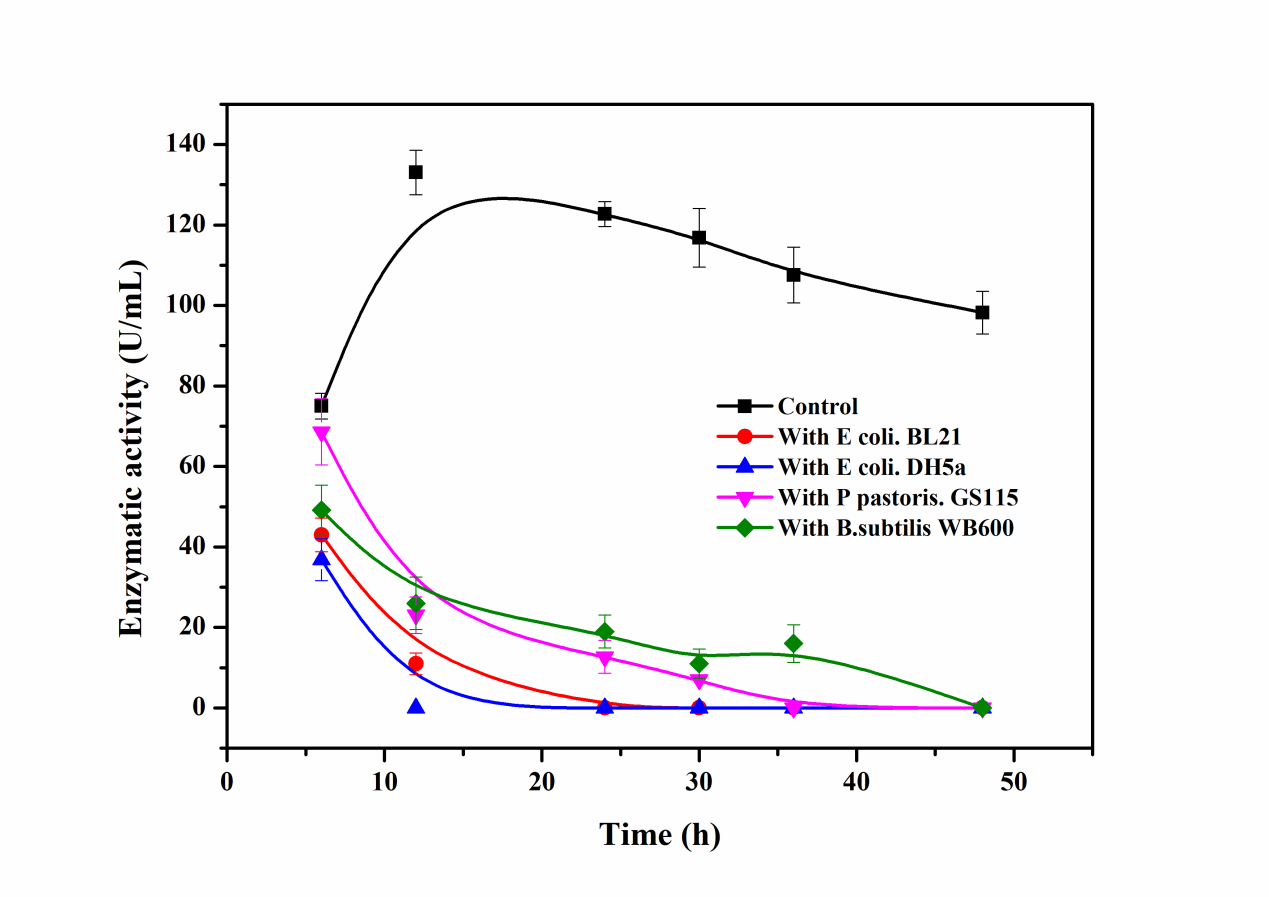


Co-culture fermentation of *R. equi* CGMCC14861 and other strains for ReCDA production.

# Supplementary Fig. S4


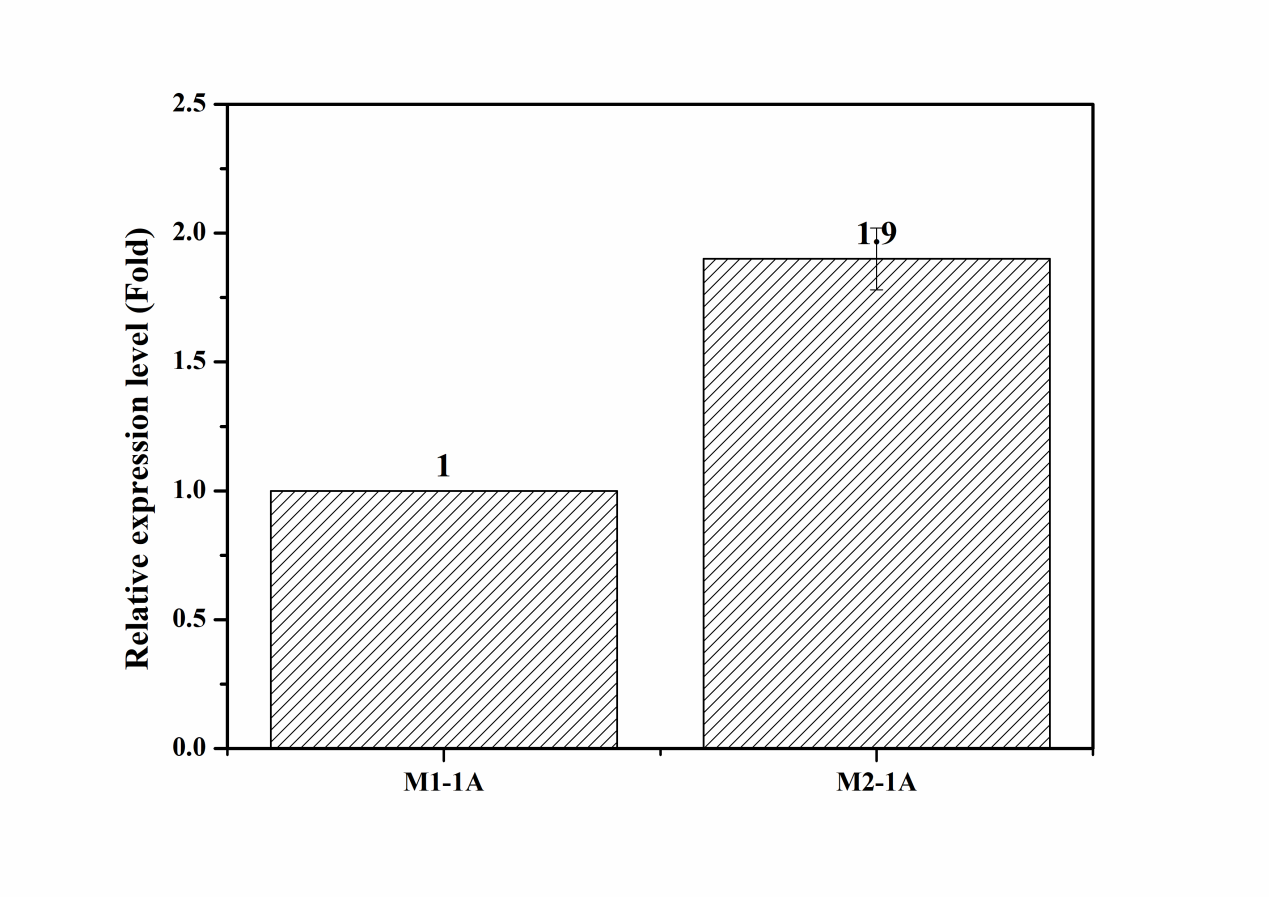
Comparison of Reverse transcription PCR of M1-1A and M2-1A. The qRT-PCR analysis was mainly performed using a Applied Biosystems 7500 Real-Time PCR System.

# Supplementary Fig. S5


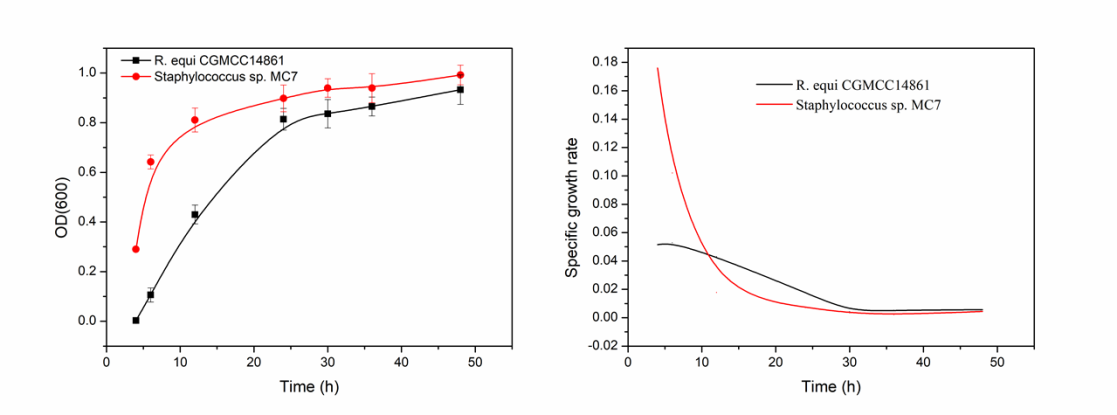


The growth profile and specific growth rate of both cultures.
